# Selections from Favorite Prescriptions of Living American Practitioners

**Published:** 1859-03

**Authors:** 


Art. IX.—*Selections from Favorite Prescriptions of Living American Practitioners.* By Horace Green, M.D., Emeritus Professor of the Theory and Practice of Medicine in the New York Medical College, etc. 8vo., pp. 206. Willey & Halsted: New York. 1858.

From the title of this work we had expected to find a more diversified collection of formulæ from the majority of American physicians, inasmuch as Dr. Green was, as he states, in the habit of being visited annually by nearly one thousand practitioners from different parts of the country, from whom he gleaned the basis of the volume before us. Some of the prescriptions are not American, and should, therefore, have been excluded; and some of their authors are dead, so that we have a right to find fault with the title of the work. These formulæ are arranged under their proper heads and many, no doubt, are useful and are capable of fulfilling the indications for which they are intended. We would, however, caution the young practitioner from using any particular formulæ for any one disease, merely because the diagnosis is made out and such a one is recommended. We enter our protest against works of this nature being placed in their hands, as they generally do more harm than good.

